# Embryonic Hypotaurine Levels Contribute to Strain-Dependent Susceptibility in Mouse Models of Valproate-Induced Neural Tube Defects

**DOI:** 10.3389/fcell.2022.832492

**Published:** 2022-02-21

**Authors:** John W. Steele, Ying Linda Lin, Nellie Chen, Bogdan J. Wlodarczyk, Qiuying Chen, Nabeel Attarwala, Madhu Venkatesalu, Robert M. Cabrera, Steven S. Gross, Richard H. Finnell

**Affiliations:** ^1^ Center for Precision Environmental Health, Department of Molecular and Cellular Biology, Baylor College of Medicine, Houston, TX, United States; ^2^ Department of BioSciences, Rice University, Houston, TX, United States; ^3^ Department of Pharmacology, Weill Cornell Medical College, New York, NY, United States; ^4^ Department of Molecular and Human Genetics, Baylor College of Medicine, Houston, TX, United States; ^5^ Department of Medicine, Baylor College of Medicine, Houston, TX, United States

**Keywords:** valproate, valproic acid, neural tube defect, antiseizure medication, anticonvulsant, antiepileptic, epilepsy

## Abstract

Valproic acid (VPA, valproate, Depakote) is a commonly used anti-seizure medication (ASM) in the treatment of epilepsy and a variety of other neurological disorders. While VPA and other ASMs are efficacious for management of seizures, they also increase the risk for adverse pregnancy outcomes, including neural tube defects (NTDs). Thus, the utility of these drugs during pregnancy and in women of childbearing potential presents a continuing public health challenge. Elucidating the underlying genetic or metabolic risk factors for VPA-affected pregnancies may lead to development of non-teratogenic ASMs, novel prevention strategies, or more targeted methods for managing epileptic pregnancies. To address this challenge, we performed unbiased, whole embryo metabolomic screening of E8.5 mouse embryos from two inbred strains with differential susceptibility to VPA-induced NTDs. We identified metabolites of differential abundance between the two strains, both in response to VPA exposure and in the vehicle controls. Notable enriched pathways included lipid metabolism, carnitine metabolism, and several amino acid pathways, especially cysteine and methionine metabolism. There also was increased abundance of ω-oxidation products of VPA in the more NTD-sensitive strain, suggesting differential metabolism of the drug. Finally, we found significantly reduced levels of hypotaurine in the susceptible strain regardless of VPA status. Based on this information, we hypothesized that maternal supplementation with L-carnitine (400 mg/kg), coenzyme A (200 mg/kg), or hypotaurine (350 mg/kg) would reduce VPA-induced NTDs in the sensitive strain and found that administration of hypotaurine prior to VPA exposure significantly reduced the occurrence of NTDs by close to one-third compared to controls. L-carnitine and coenzyme A reduced resorption rates but did not significantly reduce NTD risk in the sensitive strain. These results suggest that genetic variants or environmental exposures influencing embryonic hypotaurine status may be factors in determining risk for adverse pregnancy outcomes when managing the health care needs of pregnant women exposed to VPA or other ASMs.

## 1 Introduction

Valproic acid (VPA), marketed as Depakene and Depakote among others, is used widely as an anticonvulsant, a mood stabilizer, a prophylactic, and is used in some cases for treatment of schizophrenia (Wlodarczyk et al., 2012). It was first approved for medical use in the United States in 1978 as an anti-seizure medication (ASM) and has proven effective against multiple types of seizures (Scott, 1993; Marson and Sills, 2015). There are between 7.6 and 12.7 million epileptic women in the United States with close to 25,000 epileptic pregnancies each year (Meador et al., 2008; Harden et al., 2009), but managing epilepsy during pregnancy can be challenging given the maternal physiologic changes that can alter pharmacodynamics and effectiveness of ASMs (Sabers and Tomson, 2009; Vajda, 2012). Moreover, the number of women exposed to VPA or ASMs during pregnancy is even greater given the wide range of uses for these drugs apart from epilepsy management. It is firmly established that *in utero* ASM exposure greatly enhances risk for adverse birth outcomes, with most studies reporting an approximately two to three-fold increased risk of major congenital malformations in ASM-treated epileptic pregnancies compared to epileptic pregnancies that went untreated or those of non-epileptic women (Tomson and Battino, 2009; Tomson and Battino, 2012). Common congenital anomalies observed from ASM exposures include congenital heart defects, hypospadias, facial clefts, growth retardation, microcephaly, and neural tube defects (NTDs) (Wlodarczyk et al., 2012). Prenatal VPA exposure has also been linked to intellectual disabilities and delayed childhood milestones (Daugaard et al., 2020).

A prospective dose-dependent study on the effects of VPA and other ASMs, the Neurodevelopmental Effects of Antiepileptic Drugs (NEAD) study, determined that adverse pregnancy outcomes were observed in 20.3% of VPA-treated pregnancies (Meador et al., 2006), while major congenital anomalies occur at a rate as high as 8.7%, considerably higher than other ASMs (Li et al., 2021). In particular, it appears VPA is associated with a 10–20-fold increased risk for having an NTD-affected pregnancy (Pennell, 2006). NTDs are a family of severe birth defects resulting from failed morphogenesis of the embryonic precursor to the brain and spinal cord, the neural tube. They have a complex underlying etiology derived from a multitude of dynamically interacting genetic and environmental factors (Wallingford et al., 2013). The teratogenic mechanism by which VPA causes NTDs and other congenital anomalies remains an unanswered question. Preventing these birth defects while still enabling women of childbearing potential to safely benefit from the therapeutic properties of these drugs requires us to understand the underlying risk factors and mechanisms of action.

There are undoubtedly genetic factors predisposing mothers and infants to ASM-induced teratogenic risk. Studies in mice have repeatedly demonstrated that VPA sensitivity is strain-dependent suggesting a strong genetic contribution. It was shown in the 1980s that a daily exposure to 600 mg/kg VPA in the Swiss-Vancouver (SWV) mouse strain between E6.5 and E8.5 produced exencephaly phenotypes in 82% of embryos, while no affected embryos were observed with the same exposure in C57BL/6J (C57) (Finnell and Chernoff, 1985). Even when this experiment was repeated under *ex vivo* conditions using whole embryo roller culture, SWV embryos were still up to three times more sensitive to developmental toxicity and the teratogenic effects of VPA than those of C57, suggesting that at least some of the underlying mechanisms contributing to differential strain sensitivity are embryonic and not maternal (Naruse et al., 1988). Another 1980s study showed differential sensitivity to VPA-induced NTDs between DBA/2J, LM/BC, and SWV strains, with SWV again being the most sensitive (Finnell et al., 1988). Several attempts have been made to discern underlying factors contributing to VPA susceptibility in the SWV strain, which is sometimes referred to as SWV-Fnn. Gene expression analysis revealed strain-dependent alterations in folate and one-carbon metabolism pathway genes (Finnell et al., 1997), and genomic fine mapping has been reported on in several publications over the last 2 decades, where intercrossing the C57 and SWV-Fnn strains led to identification of a candidate sensitivity locus (Beck, 1999; Finnell et al., 2000; Beck, 2001; Lundberg et al., 2004; Taiwo et al., 2020). Notably, the sensitivity locus contained all but one member of the acyl-CoA synthetase-medium chain (ACSM) family of genes, the exception being the gene encoding ACSM4. ACSM enzymes participate in the drug metabolism of VPA by producing valproyl-CoA which is subsequently driven into mitochondrial β-oxidation (Silva et al., 2008). Thus, historical attempts to identify what contributes to VPA sensitivity in this strain have pointed at a metabolic mechanism.

Given the extensive metabolic activity of VPA and its constituent metabolites, a metabolism-dependent mechanism of VPA sensitivity is highly plausible. Therefore, we performed whole embryo metabolomics on E8.5 embryos from C57BL/6J and SWV-Fnn with and without VPA exposure. The objective was to identify strain-dependent alterations in metabolism, either inherent in the native metabolome of SWV embryos or induced in response to VPA exposure, that could explain their increased predisposition for VPA-inflicted NTDs. We then used this information to devise and employ novel mitigation strategies in the form of maternal supplements that could reduce developmental toxicity phenotypes in SWV embryos after VPA exposure.

## 2 Methods

### 2.1 Whole Embryo Metabolomic Profiling

#### 2.1.1 VPA Treatment and Embryo Collection

C57BL/6J (C57) mice were obtained from Baylor College of Medicine’s (BCM) Center for Comparative Medicine, while SWV-Fnn (SWV) mice are maintained by the Finnell Laboratory at BCM. Pregnancy was determined through timed mating and confirmed by weighing pregnant dams. On gestational day 8.5, pregnant C57 or SWV dams received a 600 mg/kg intraperitoneal (IP) injection of VPA (sodium salt) (Sigma) dissolved in sterile water for injection, USP (Hospira), or a vehicle injection. All injections were conducted at a volume of 10 ml/kg. Two to four dams were utilized for each treatment group to capture potential inter-litter variability. Embryo samples were collected 2 h post injection. Dams were euthanized by cervical dislocation followed by surgical creation of bilateral pneumothorax to avoid metabolic alterations that may be induced by other forms of euthanasia. Embryos were quickly separated from their yolk sacs in sterile PBS, washed briefly in sterile water, snap frozen in liquid nitrogen, and stored at −80°C until metabolite extraction.

#### 2.1.2 Metabolite Extraction and LC-MS

Metabolites from the E8.5 embryos were extracted using an 80:20 methanol:water mixture chilled by dry ice, and the extracts were homogenized by bead-beating for 45 s with a Tissuelyser cell disrupter (Qiagen). The samples were centrifuged at 4°C for 5 min at 5,000 rpm, and the supernatant was reserved at −80°C while the extraction procedure was repeated on each debris pellet two additional times. The final debris pellet was reserved for protein quantification for normalization purposes, while the supernatants from each repeated extraction were pooled for each individual sample, and the solvent was evaporated on low in a vacuum centrifuge at 4°C. The dried extracts were reconstituted in 70% acetonitrile at a relative protein concentration of 1 μg/ml. In total, 5 μl of each sample was injected for LC-MS-based profiling using an Agilent Model 1290 Infinity II liquid chromatograph coupled to an Agilent 6550 iFunnel time-of-flight mass spectrometer utilizing aqueous normal phase (ANP) chromatography on a Diamond Hydride column (Microsolv). Mobile phases consisted of: (A) 50% isopropanol containing .025% acetonitrile and (B) 90% acetonitrile containing 5 mM ammonium acetate. In total, 6 μM EDTA was added to the mobile phase to eliminate metal ion interference with chromatograph peak integrity and electrospray ionization. The following gradient was applied: 0–1.0 min, 99% B; 1.0–15.0 min, 20% B; 15.0–29.0, 0% B; 29–37 min, 99% B. Metabolite measurements were normalized using flanking quality control samples prepared from a pool of all samples, which were run every six injections.

#### 2.1.3 Metabolomics Data Analysis

The raw data were analyzed using MassHunter Profinder 8.0 and MassProfiler Professional (MPP) 15.1 software (Agilent). Metabolite structures were identified using an in-house annotated metabolite database created using MassHunter PCDL manager 8.0 (Agilent) based on monoisotopic neutral masses (<5 ppm mass accuracy) and chromatographic retention times. A molecular formula generator (MFG) algorithm in MPP was used, based on weighted consideration of monoisotopic mass accuracy, isotope abundance ratios, and spacing between isotope peaks. A tentative compound ID was assigned when MFG scores concurred with the PCDL database for a given candidate molecule. Tentatively assigned molecules were verified based on a match of LC retention times and/or MS-MS fragmentation spectra for pure molecule standards contained in the in-house database. Common R packages such as omu (https://cran.r-project.org/web/packages/omu) and Metaboanalyst (https://www.metaboanalyst.ca) were used for data analysis and visualization, including hierarchical clustering, pathway enrichment analysis, principal component, and partial least squares-discriminant analyses. For analysis using Metaboanalyst, non-informative features were filtered out using the interquantile range method. Missing values were estimated feature-wise using a k-nearest neighbors (KNN) algorithm. Samples were normalized by median, and the dataset was log-transformed and mean-centered.

### 2.2 Maternal Supplementation With L-Carnitine, Coenzyme A, and Hypotaurine

#### 2.2.1 Treatment and Embryo Collection

Maternal supplementation experiments were only performed in SWV mice, as that is the sensitive strain of interest. On gestational day 8.5, pregnant dams received their assigned supplement *via* IP injection or a vehicle control injection at a volume of 5 ml/kg, followed within 15 min by IP injection with VPA (600 mg/kg) also at a volume of 5 ml/kg, such that the entire volume of injected diluent was 10 ml/kg. All supplements were dissolved in sterile water for injection, USP, and the control group received only the sterile injection water during the first injection, yet still received the 600 mg/kg VPA injection since the goal was to determine how well the supplements reduced developmental toxicity after VPA exposure. L-Carnitine (Cayman Chemical), coenzyme A (Cayman Chemical), and hypotaurine (Sigma) were administered at 400, 200, and 350 mg/kg, respectively. Embryos were collected between E11.5 and E13.5 for assessment of exencephaly phenotypes. Prior to collection dams were euthanized by CO_2_ asphyxiation followed by cervical dislocation. Embryos were scored based on whether an exencephaly phenotype was observed and resorptions were also counted. The embryos were imaged, and some were reserved for sex determination by PCR amplification targeting SRY (Forward Primer: 5′-TTG​TCT​AGA​GAG​CAT​GGA​GGG​CCA​TGT-3′; Reverse Primer: 5′-CAC​TCC​TCT​GTG​ACA​CTT​TAG​CCC​TCC​GA-3′, Integrated DNA Technologies).

#### 2.2.2 Statistical Analyses

Two-sample proportion tests were conducted using Rcmdr (https://cran.r-project.org/web/packages/Rcmdr) to determine whether the supplement reduced exencephaly, resorption rates, or overall developmental toxicity compared to the control group (*α* = .05). Type II error probability (*β*) associated with significant observations is also reported, and observations are only accepted with confidence where *β* > .80. We also followed up these observations by comparing occurrences of exencephaly or resorptions and percentage of affected vs. unaffected embryos per litter. In those cases, statistical significance was determined by one-way ANOVA with Dunnett’s multiple comparisons test or two-way ANOVA with Šídák’s multiple comparisons test, depending on the conditions of the test. These tests were performed using Prism (Graphpad).

## 3 Results

### 3.1 Strain-Dependent Susceptibility of Swiss-Vancouver Embryos to Valproic Acid

**FIGURE 1:**
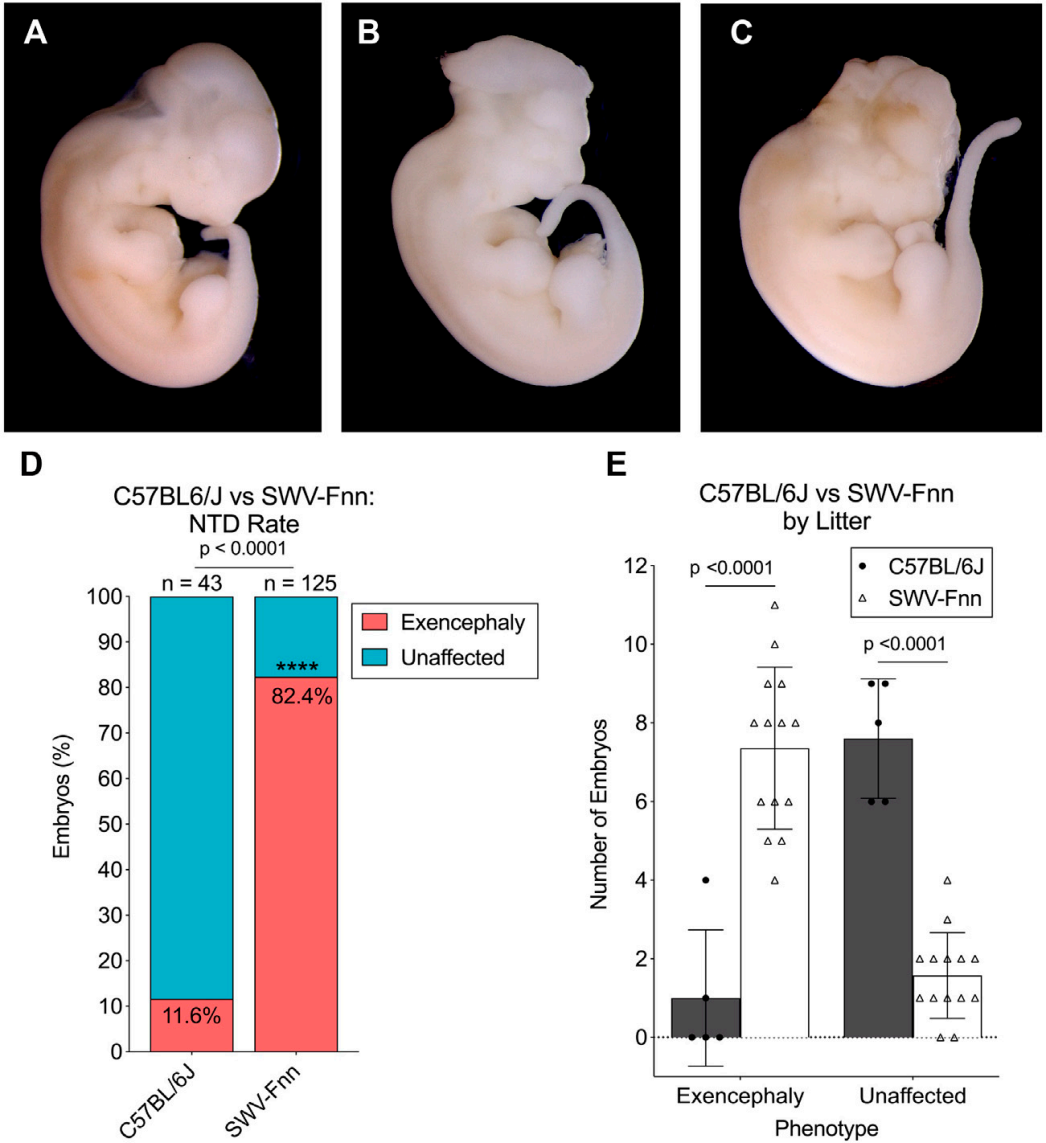
Strain-dependent susceptibility to VPA confirmed in SWV embryos. **(A)** Image of unaffected E12.5 SWV embryo exposed to 600 mg/kg VPA, appearing developmentally normal. **(B,C)** Two examples of E12.5 SWV embryos presenting exencephaly phenotypes after exposure to VPA (600 mg/kg). **(D)** Proportion of embryos with exencephaly phenotypes in C57BL/6J (C57) and SWV-Fnn (SWV) litters exposed to 600 mg/kg VPA. *Statistical significance determined by two-sample proportion test (*α* < .05). **(E)** Number of exencephalic versus unaffected embryos in each C57 or SWV litter exposed to 600 mg/kg VPA. *Statistical significance determined by two-way ANOVA with Šídák’s multiple comparisons (*α* < .05).

We verified the sensitivity of the SWV strain to VPA-induced developmental toxicity in comparison to C57, both confirming the findings of previously published literature and ensuring that our experimental rationale was valid. Over the course of this study, 125 SWV embryos from 14 separate litters were observed for exencephaly phenotypes after a 600 mg/kg maternal exposure to VPA on E8.5. Examples of exencephalies observed in SWV embryos compared to unaffected embryos are demonstrated in Figures 1A–C. We also observed 43 C57 embryos from 5 separate litters after receiving the same dose. Between E11.5 and E13.5, 103 of 125 SWV embryos had exencephaly (82.4%), while only 5 of 43 C57 embryos had exencephaly (11.6%, *p* < .0001) (Figure 1D). Only 2 of 5 C57 litters were affected, and the average number of exencephalic embryos per litter was only 1.0, while all 14 SWV litters were affected with an average of 7.4 exencephalic embryos per litter (*p* < .0001) (Figure 1E). While SWV litter sizes are slightly larger than those of C57, an average of 7.6 embryos did not have any NTD phenotypes in C57 litters, and only about 1.5 embryos did not have NTD phenotypes in the average SWV litter (*p* < .0001) (Figure 1E).

We also tested whether there was a sex-dependent effect on susceptibility to NTD phenotypes in VPA-treated SWV by genotyping a sample of 106 embryos for the presence of the SRY gene, a gene located on the Y-chromosome. The subsample consisted of 52 females and 54 males. 83.3% of male embryos and 82.7% of female embryos developed exencephaly (Supplementary Figure S1). Thus, the mechanisms of VPA susceptibility in SWV were not dependent on embryonic sex.

### 3.2 Whole Embryo Metabolomics: Swiss-Vancouver and C57

Metabolomic profiling of E8.5 SWV and C57 embryos with and without VPA exposure was conducted to identify potential metabolic factors that could explain enhanced sensitivity of SWV embryos. The embryos were collected from euthanized dams 2 h post exposure to VPA or a vehicle injection. LC-MS was performed on 42 C57 embryos (12 from the untreated vehicle group and 30 from the VPA group) and 39 SWV embryos (15 from the untreated vehicle group and 24 from the VPA group). Three embryos were excluded from analysis (one from the SWV VPA group, one from the C57 VPA group, and one from the C57 untreated group) when it was discovered they shared an anomalous group of missing features compared to the other embryos. We identified 723 distinct metabolite peaks, with about 613 metabolites ultimately contributing to the analysis after data filtering for non-informative variables.

#### 3.2.1 Effects of Valproic Acid on the Embryonic Metabolome

We first analyzed the effects of VPA on the global embryonic metabolome by comparing the untreated vehicle embryos to the VPA group regardless of strain. There were 194 metabolites of differential abundance (MDAs) (FDR < .05) detected in the VPA-treated embryos, of which 39 were increased in abundance while 155 were decreased (Supplementary Table S1). Many of the dysregulated metabolites were classified in lipid or amino acid metabolism (Supplementary Figure S2). While these two classifications account for a majority of the metabolites in the dataset, they were significantly over-represented as MDAs using Fisher’s Exact Test (lipid metabolism *p* = .0016, amino acid metabolism *p* = .0005). One notable affected pathway was carnitine metabolism. Seven carnitine species were decreased in response to VPA, including 2-hexanoylcarnitine, deoxycarnitine, linolenylcarnitine, hexanoylcarnitine, acetylcarnitine, valerylcarnitine, and carnitine itself. This is consistent with existing knowledge that metabolism of VPA depletes carnitine stores, and that long term VPA treatment or VPA overdose results in carnitine deficiency (Melegh et al., 1987; Van Wouwe, 1995; Lheureux and Hantson, 2009). Another pathway enriched with MDAs was glycine, serine, and threonine metabolism, from which guanidinoacetate and sarcosine were increased, while betaine, choline, dimethylglycine, serine, threonine, and creatine were decreased. Depletion of choline and its downstream metabolites may be the result of the disruptions observed in glycerophospholipid metabolism, since choline is generally stored in the form of phosphatidylcholines and sphingomyelins, which were represented by quite a few of the MDAs in the glycerophospholipid classification. These metabolites also play an important role in providing methyl groups to the methionine cycle for turnover of homocysteine. Cysteine and methionine metabolism were indeed perturbed in the VPA-treated embryos. Abundance of methionine and s-adenosylmethionine (SAM) were decreased, while homocysteine and s-adenosylhomocysteine (SAH) were increased, leading to an overall decrease in the SAM/SAH ratio (Supplementary Figure S3). The SAM/SAH ratio describes the capacity of these embryos to perform the myriad of methylation reactions across their global metabolic landscape, including in the synthesis of other metabolites or for epigenetic modifications. For example, this decrease in the SAM/SAH ratio likely explains the decrease in creatine and increase in guanidinoacetate, since SAM is required to synthesize creatine from its guanidinoacetate precursor. While creatine supplementation in third trimester human pregnancies has been explored (Dickinson et al., 2014), very little is known about the role of creatine in early mammalian embryos, such as in the developmental timepoint under study here. While creatine can serve as a source of rapid energy mobilization within cells, and rapidly growing embryos where most cells are proliferating and differentiating may be an environment with high cellular energy demands, not enough is known about requirements for creatine in developing embryos to assess whether creatine depletion causes developmental toxicity. However, the importance of the methionine cycle during this timepoint of development is well known. High levels of homocysteine are associated with increased risk for NTDs, and exposure to drugs that impair the methylation cycle, such as methotrexate, are also known to cause NTDs and other defects. So, these observations may offer some explanation to the general developmental toxicity of VPA. It should also be noted that we observed evidence of oxidative stress in the VPA-treated embryos, as the ratio of oxidized glutathione to reduced glutathione was increased in response to VPA (Supplementary Figure S4). That said, the observed disruptions to methylation metabolism and oxidative metabolism were common to both SWV and C57 embryos and are, therefore, not likely explanatory for enhanced VPA susceptibility in the SWV strain.

#### 3.2.2 Strain-specific Metabolic Responses in Swiss-Vancouver and C57

**FIGURE 2:**
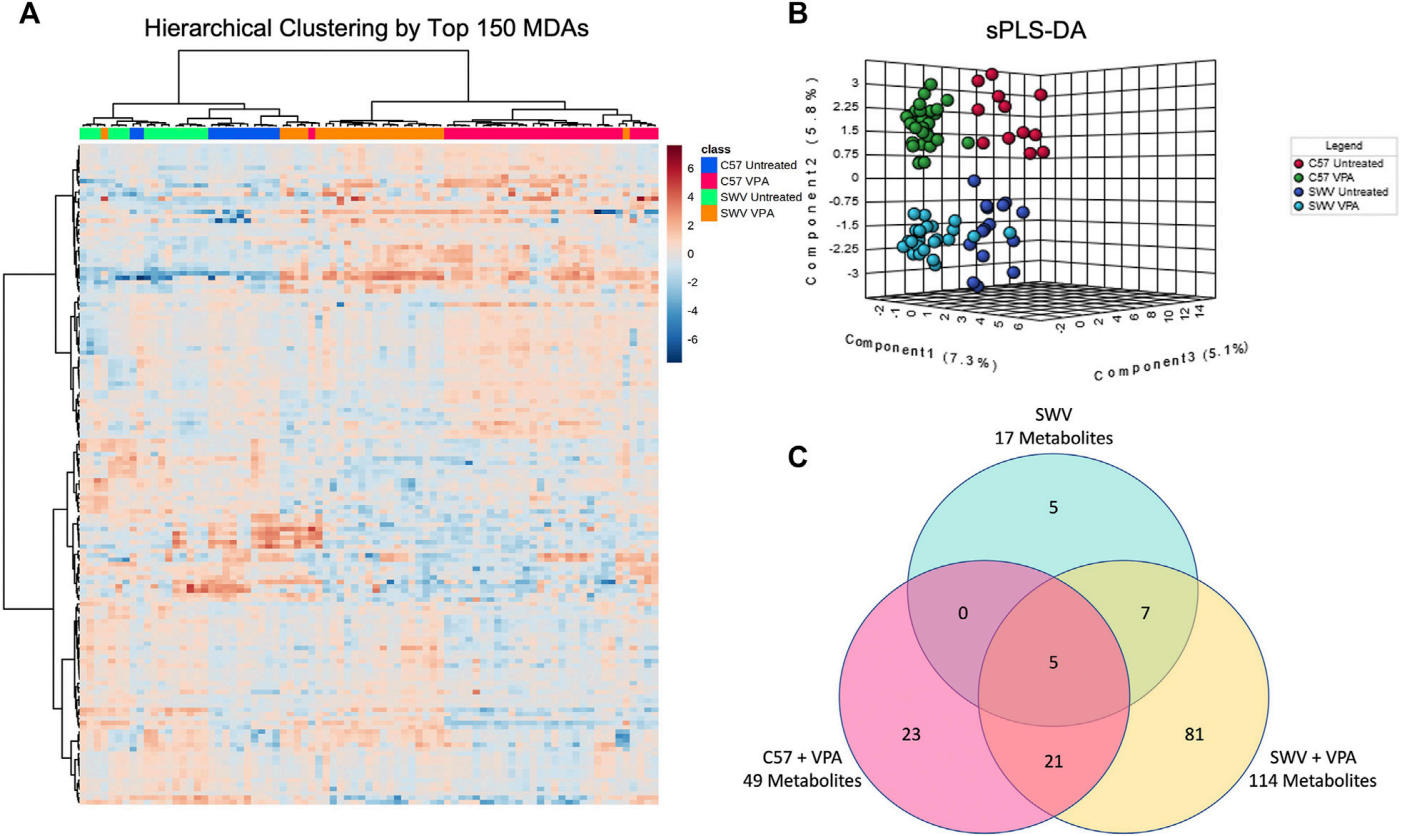
Strain-dependent metabolic signatures associated with VPA exposure. **(A)** Untargeted hierarchical clustering based on 150 highest ranked metabolites of differential abundance (MDAs) after FDR correction. Metabolites are clustered in rows, while columns represent individual embryos. Each column is marked with a colored bar signifying the classification of the corresponding embryos by strain and VPA treatment group. The row and column dendrograms represent clustering by Pearson correlation using Ward’s linkage method. **(B)** Metaboanalyst sparse partial least squares discriminant analysis (sPLS-DA) demonstrating distinctive clustering of embryos by treatment and strain. **(C)** Venn-diagram displaying number of MDAs in SWV, C57 VPA, and SWV VPA embryos compared to untreated C57 controls.

Pearson hierarchical clustering of the top 150 MDAs revealed that embryos clustered most strongly by VPA treatment status, however they also clustered by mouse strain indicating that there were indeed distinctive metabolic signatures differentiating SWV and C57 (Figure 2A). Sparse partial least squares discriminant analysis (sPLS-DA) also suggested that metabolic signatures could be classified by strain and VPA treatment status based on embryos clustering according to these respective categories (Figure 2B). We therefore sought to identify which features distinguished SWV from C57 embryos. Using the untreated vehicle C57 embryos as a baseline, we looked for MDAs in the untreated SWV (Supplementary Table S2), the C57 VPA (Supplementary Table S3), and SWV VPA (Supplementary Table S4). We identified 17 MDAs in SWV compared to C57, 12 of which were exclusive to SWV or SWV VPA samples (Figure 2C). Notably, VPA-treated SWV showed the greatest metabolomic departure from untreated C57, with 114 identified MDAs compared to only 49 MDAs distinguishing VPA-treated C57 from untreated C57 (Figure 2C). Thus, the global metabolic response to VPA in SWV appears more sensitive overall.

**FIGURE 3:**
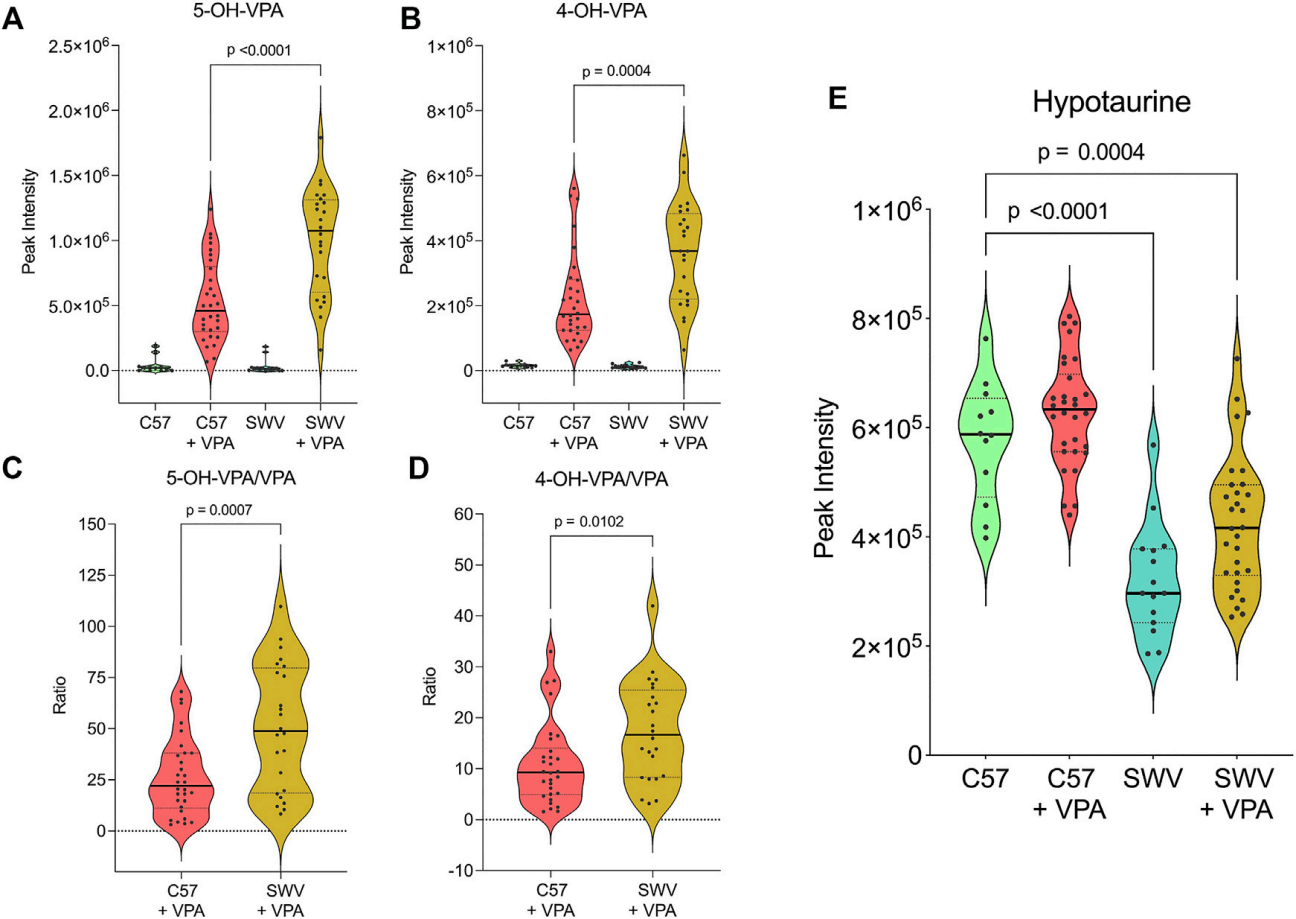
Metabolites of interest distinguishing SWV and C57 embryos. Metabolite peak intensities for **(A)** 5-OH-VPA and **(B)** 4-OH-VPA. **(C)** Ratio of 5-OH-VPA to unmetabolized VPA in VPA-treated embryos. **(D)** Ratio of 4-OH-VPA to unmetabolized VPA in VPA-treated embryos. **(E)** Metabolite peak intensities for hypotaurine. *Statistical significance determined by Student’s t-test **(C,D)** and one-way ANOVA with Tukey’s multiple comparisons **(A,B,E)** (*α* < .05).

When comparing MDAs between VPA-treated SWV and VPA-treated C57, two metabolites of VPA were identified as having differential abundance, 5-OH-VPA and 4-OH-VPA. Both metabolites are ω-oxidation products of VPA metabolism, and both were higher in SWV embryos 2 h post exposure (Figures 3A,B). We compared the ratio of these ω-oxidation products to unmetabolized VPA and found the ratio to be significantly elevated in SWV for both 5-OH-VPA (*p* = .0007) and 4-OH-VPA (*p* = .0102) (Figures 3C,D). These data would suggest that SWV are metabolizing VPA differently than C57, either at a different rate or to different downstream fates.

Many MDAs differentiating SWV from C57 embryos were glycerophospholipids, including phosphatidylcholines and sphingomyelins (Supplementary Table S2). Since VPA appeared to dramatically impact the profile of these lipids, it may be the case that the lipid profile of SWV embryos make them more susceptible to the alterations in lipid-choline metabolism induced by VPA. However, given the complexity and sheer diversity of these metabolites, it is difficult to effectively analyze this hypothesis. That said, one notable MDA identified in SWV that strongly distinguished them from C57 was hypotaurine, which was strikingly decreased in abundance regardless of VPA-treatment status (Figure 3E). Hypotaurine is a sulfinic acid intermediate in the metabolism of cysteine and homocysteine. Given the apparent differences in this metabolic pathway observed in response to VPA, we postulated that the apparent deficiency of hypotaurine in SWV embryos compared to C57 may be explanatory for their enhanced susceptibility to VPA.

### 3.3 Maternal Supplements as Potential Interventions

While hundreds of MDAs were ultimately identified, we chose to focus on the two primary findings that distinguished SWV from C57 embryos: 1) the differential abundance of VPA metabolites, specifically the ω-oxidation products, and 2) the decreased abundance of hypotuarine. We hypothesized that one or both of these differences in metabolism may explain the sensitivity of SWV embryos to VPA-induced developmental toxicity, especially with regards to NTD phenotypes. We tested these hypotheses using maternal co-supplements, which we expected may decrease the occurrence of NTDs, the number of resorptions, or overall developmental toxicity of VPA.

#### 3.3.1 Effect of L-Carnitine and Coenzyme A Supplementation on Valproic Acid Susceptibility in Swiss-Vancouver

**FIGURE 4:**
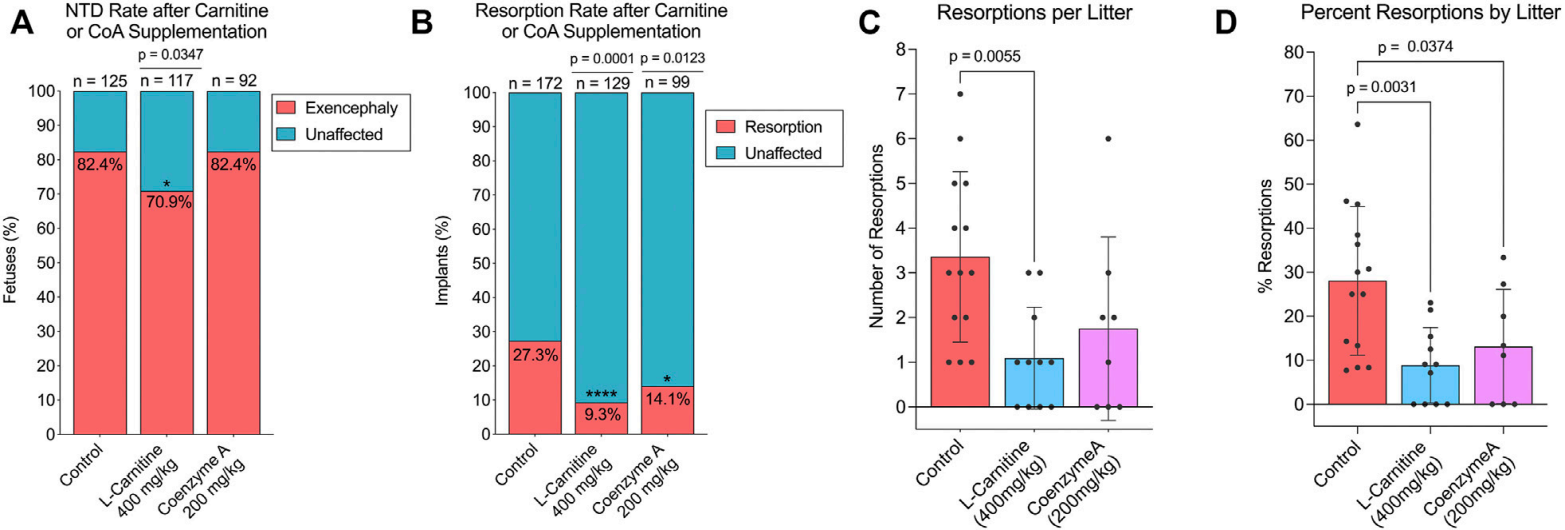
Effect of L-carnitine or coenzyme A supplementation on NTD and resorption rates in VPA-treated SWV. **(A)** Proportion of embryos with exencephaly phenotypes observed in the placebo, L-carnitine (400 mg/kg), and coenzyme A (200 mg/kg) groups. **(B)** Proportion of resorption phenotypes observed in the placebo, L-carnitine (400 mg/kg), and coenzyme A (200 mg/kg) groups. **(C)** Number of resorptions observed per litter in the placebo, L-carnitine (400 mg/kg), and coenzyme A (200 mg/kg) groups. **(D)** Percent resorptions observed per litter in the placebo, L-carnitine (400 mg/kg), and coenzyme A (200 mg/kg) groups. *Statistical significance determined by individual two-sample proportion tests comparing the carnitine or CoA groups to the control group **(A,B)** or one-way ANOVA with Dunnett’s multiple comparisons **(C,D)** (*α* < .05).

Since our data suggested that SWV may be favoring ω-oxidation of VPA over β-oxidation, we hypothesized that supplementation with carnitine or CoA may reduce developmental toxicity phenotypes in SWV embryos exposed to VPA by driving more VPA to β-oxidation fates. We tested this hypothesis by administering 400 mg/kg L-carnitine or 200 mg/kg CoA *via* IP injection to pregnant dams on E8.5, shortly before a 600 mg/kg injection of VPA. Dams were euthanized between E11.5 and E13.5 to assess developmental toxicity phenotypes, primarily the number of resorptions and the occurrence of NTDs. It should be noted that in this strain, we only observe exencephaly phenotypes and not spina bifida in response to VPA. Outcomes were compared to a control group that received a vehicle injection of sterile injection water in place of L-carnitine or CoA. For the control group, which only received VPA, 172 implants were observed across 14 litters. There were 47 resorptions (27.33%), and of 125 non-resorbed embryos, 103 presented with exencephaly (82.4%). We did not observe a reduction in NTD phenotypes in the CoA-supplemented group (70 NTDs/85 embryos, 82.35%), but did see a slight reduction for NTDs in the carnitine-supplemented group (83 NTDs/117 embryos, 70.94%, *p* = .0347) (Figure 4A; Table 1). There was a dramatic reduction in resorption rates for carnitine-supplemented litters (12 resorptions/129 implants, 9.30%, *p* < .0001), and a slightly less, but still significant reduction for CoA-supplemented litters (14 resorptions/99 implants, 14.14%, *p* = .0123) (Figure 4B; Table 2). The average number of resorptions per litter was lower in the carnitine-supplemented group (*p* = .0055) (Figure 4C), and the overall proportion of resorptions per litter was lower in both carnitine and CoA groups (*p* = .0031 and .0374, respectively) (Figure 4D). To assess an overall reduction in developmental toxicity, we combined resorptions and NTDs as total affected embryos. For the control group, 150 of 172 observations fell into this category (87.21%). Overall developmental toxicity was decreased in the group receiving 400 mg/kg carnitine (95/129, 73.64%, *p* = .0028), but not in the group receiving CoA (84/99, 84.85%, *p* = .5857) (Table 2). Thus, these data suggest that L-carnitine co-supplementation may reduce overall developmental toxicity associated with VPA susceptibility in SWV, primarily through reduction in resorptions and a small reduction in NTDs. We also tested a smaller group of dams at an 800 mg/kg dose of L-carnitine and did not observe a dose response regarding reduction in developmental toxicity, resorptions, or NTDs (Tables 1, 2). While CoA supplementation reduced resorption rates, the overall developmental toxicity was not significantly decreased (Table 2).

**TABLE 1:** Outcome of maternal supplements on VPA phenotypes (embryos).

Group	Litters	Embryos	Exencephaly (%)	Unaffected (%)	*p*-value (*β*)
Control	14	125	103 (82.40%)	22 (17.60%)	
L-Carnitine 400 mg/kg	11	117	83 (70.94%)	34 (29.06%)	**.0347** (.55)
L-Carnitine 800 mg/kg	6	57	44 (77.19%)	13 (22.81%)	.4084 (.47)
Coenzyme A 200 mg/kg	8	85	70 (82.35%)	15 (17.65%)	.9930 (.00)
Hypotaurine 350 mg/kg	10	92	**54 (58.70%)**	**38 (41.30%)**	*.0001 *(.95)

p-values less than .05 and β values greater than .80 are highlighted with bold font.

**TABLE 2:** Outcome of maternal supplements on VPA phenotypes (all implants).

Group	Litters	Implants	Resorptions (%)	*p*-value (*β*)	Total affected NTD + resorptions (%)	*p*-value
Control	14	172	47 (27.33%)		150 (87.21%)	
L-Carnitine 400 mg/kg	11	129	**12 (9.30%)**	*<.0001 *(.97)	**95 (73.64%)**	*.0028
L-Carnitine 800 mg/kg	6	70	13 (18.57%)	.1527 (.23)	57 (81.43%)	.2463
Coenzyme A 200 mg/kg	8	99	14 (14.14%)	**.0123** (.63)	84 (84.85%)	.5857
Hypotaurine 350 mg/kg	10	92	21 (18.58%)	.0903 (.35)	**75 (66.37%)**	*<.0001

p-values less than .05 and β values greater than .80 are highlighted with bold font.

#### 3.3.2 Effect of Hypotaurine Supplementation on Valproic Acid Susceptibility in Swiss-Vancouver

**FIGURE 5:**
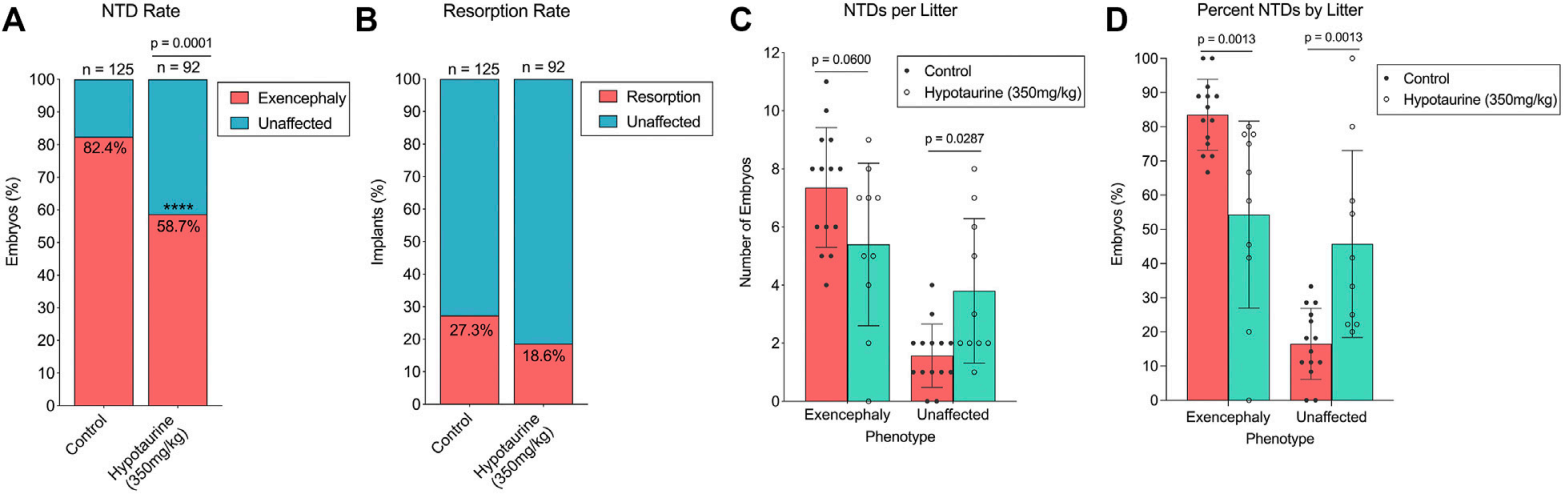
Effect of hypotaurine supplementation on NTD and resorption rates in VPA-treated SWV. **(A)** Proportion of embryos with exencephaly phenotypes observed in the placebo and hypotaurine (350 mg/kg) groups. **(B)** Proportion of resorption phenotypes observed in the placebo and hypotaurine (350 mg/kg) groups. **(C)** Number of NTDs observed per litter in the placebo and hypotaurine (350 mg/kg) groups. **(D)** Percent NTDs observed per litter in the placebo and hypotaurine (350 mg/kg) groups. *Statistical significance determined by two-sample proportion tests **(A,B)** or two-way ANOVA with Šídák’s multiple comparisons **(C,D)** (*α* < .05)*.*

Given the reduced abundance of hypotaurine in SWV embryos compared to those of C57, we hypothesized that hypotaurine may have a protective effect against VPA’s teratogenic activities, and that by supplementing dams with hypotaurine prior to VPA exposure, we could reduce the occurrence of NTDs or other developmental toxicity phenotypes. We tested this hypothesis by administering 350 mg/kg hypotaurine *via* IP injection to pregnant dams on E8.5, shortly before a 600 mg/kg injection of VPA. The experimental design was similar to that of the carnitine and CoA co-supplementation experiments and was performed in conjunction with that study using the same control group of vehicle supplemented dams. Compared to the control group, the hypotaurine-supplemented group had a significantly lower proportion of exencephalic embryos (54 NTDs/92 embryos, 58.70%, *p* < .0001) (Figure 5A; Table 1). Unlike with carnitine and CoA, there was no significant reduction in resorption rates after hypotaurine supplementation (21 resorptions/92 implants, 18.58%, *p* = .0903) (Figure 5B; Table 2). The average number of exencephalic and unaffected embryos per litter were lower and higher, respectively, in the hypotaurine group compared to controls (Figure 5C), and the average proportion of exencephalic embryos per litter was lower in the hypotaurine group (Figure 5D). Thus, it is clear this is the result of an overall reduction in NTD rates, and not an artifact of reduced litter sizes or resorption rates. When we looked at overall developmental toxicity combining NTD and resorption phenotypes, we found the total affected embryos in the hypotaurine-supplemented group were significantly less than those of the control group (75/92, 66.37%, *p* < .0001) (Table 2). Therefore, the hypothesis that hypotaurine co-supplementation may prevent NTDs and reduce developmental toxicity associated with enhanced VPA sensitivity in SWV is strongly supported by the data.

## 4 Discussion

Despite the potential for developmental toxicity, VPA and other ASMs are still widely prescribed to women of childbearing potential. Since discontinuing use during pregnancy may also result in adverse outcomes with respect to the mother’s antiseizure treatment, there is a critical need to elucidate the mechanisms of VPA-induced teratogenesis and to understand the underlying genetic or metabolic mechanisms predisposing to susceptibility for adverse pregnancy outcomes. In doing so, mitigation strategies can be developed to make use of ASMs safer for pregnant women or women planning to become pregnant. Such knowledge could also help to develop alternative ASMs with lower teratogenic potential; and, if clinicians have a full picture regarding individual factors contributing to adverse susceptibilities, they can develop informed precision medicine strategies that target the right drug or intervention to the right mother-infant pairs.

In this study, we performed comparative metabolomics of embryos in two separate mouse strains with differential susceptibility to VPA-induced NTDs. Using this untargeted data, we developed hypotheses regarding metabolic mechanisms that could explain enhanced susceptibility of the SWV strain and attempted to target these mechanisms through supplemental interventions in pregnant dams with the aim of reducing developmental toxicity phenotypes. One of the relevant findings was the increased abundance of ω-oxidation metabolites of VPA, which suggested either an altered rate of the metabolism of the drug or differential flux driving VPA to separate metabolic fates. VPA is metabolized to several metabolic fates, usually in the liver (Lheureux and Hantson, 2009). And it has been proposed that different metabolites of VPA may be responsible for its developmental toxicity as well as its overall toxicity. For example, it is has been suggested in the literature that ω-oxidation products of VPA are more toxic than its β-oxidation metabolites (Lheureux and Hantson, 2009), although this assumption seems primarily based on the observation that carnitine co-supplementation can reduce toxic effects of VPA. β-oxidation of VPA requires attachment of coenzyme A (CoA) by ACSM enzymes to produce valproyl-CoA, which is shuttled into mitochondria via the carnitine transport system (Silva et al., 2008). Under our experimental conditions, it is unclear whether the differential metabolism of VPA in SWV and C57 is occurring predominantly through the dam’s hepatic system and transferred to the embryo, or if VPA metabolism is occurring within the embryos themselves. However, we postulate that embryonic metabolism of VPA may play a role here. as there is already published evidence to suggest that SWV embryos may metabolize VPA differently. Genomic fine mapping of exencephalic fetuses derived from SWV/C57 backcrossing identified a 33 MB sensitivity locus which contains genes coding for members of the ACSM family of enzymes involved in VPA metabolism (Taiwo et al., 2020). ACSM enzymes catalyze conversion of VPA to valproyl-coA, a critical first step required for further metabolism via mitochondrial β-oxidation. However, if genetic variants diminish ACSM activity in SWV embryos, it may result in more drug driven into alternative metabolic fates, such as ω-oxidation. Cytosolic ω-oxidation through cytochrome P450 (CYP) enzymes typically only accounts for about 10% of VPA metabolism, however variants in CYP genes have been shown to alter pharmacokinetics of VPA in epileptic patients (Sankar, 2007; Tan et al., 2010). ω-oxidation products of VPA metabolism are thought to be more hepatotoxic (Lheureux and Hantson, 2009), particularly 4-ene-VPA, which can undergo β-oxidation to form the reactive species (E)-2,4-diene VPA, which competes with the glutathione redox cycle by oxidizing NADPH (Liu et al., 2009). In fact, previous research has identified certain VPA metabolites as having similar teratogenicity as VPA itself (Finnell et al., 1988). Therefore, we reasonably hypothesized that differential metabolism of VPA may explain enhanced susceptibility in SWV, and that we could reduce developmental toxicity in this strain through targeted manipulation of the drug’s metabolism.

We proposed that maternal supplementation with L-carnitine or CoA would promote β-oxidation of the drug and reduce the amount of VPA ultimately fated to more toxic ω products. Carnitine facilitates transport of acyl-CoAs and valproyl-CoAs into the mitochondria, and high dose or long-term exposure to VPA results in depletion of carnitine (Melegh et al., 1987; Opala, 1990; Melegh et al., 1994; Van Wouwe, 1995; Castro-Gago et al., 1998). This is consistent with observations from our metabolomics data, which showed a reduction in embryonic carnitine levels after VPA exposure in both strains (Supplementary Figure S5). Carnitine supplementation is recommended in at-risk pediatric patients taking VPA to prevent carnitine deficiency, and there is some limited clinical evidence as well as some case studies suggesting efficacy of carnitine supplementation to treat acute VPA poisoning and VPA-induced hepatoxicity or hyperammonemia (Lheureux and Hantson, 2009; Alluin et al., 2011; Felker et al., 2014; Haznedar et al., 2019). Moreover, carnitine and CoA were both shown to reduce NTD rates in CD-1 mice (Nagao et al., 1996), suggesting a similar rescue in SWV could be possible, assuming this proposed mechanism of susceptibility. Ultimately, we found that L-carnitine at 400 mg/kg reduced overall developmental toxicity of VPA in SWV embryos, primarily through a major reduction in resorption rates. Although there was some minor reduction in occurrence of NTDs that was statistically significant, the Type-II error estimate was only .55 given the observed effect size, and increasing the dose to 800 mg/kg did not decrease NTD rates further. CoA supplementation at 200 mg/kg also reduced resorption rates, although not as effectively as carnitine, nor was there any observed reduction in NTD rates. These data do not support the hypothesis that increased ω-oxidation of VPA is responsible for enhance susceptibility in SWV. That said, they do not exclude this hypothesis either as it is possible carnitine and CoA supplementation did not alter VPA metabolic fate as predicted. More targeted metabolic studies would be required to address this question more accurately.

We also proposed that hypotaurine supplementation may reduce developmental toxicity or NTD phenotypes in SWV based on their apparent hypotaurine deficiency compared to C57. Our hypothesis centered on the possibility that hypotaurine may have a protective effect against the teratogenic activities of VPA, thus making SWV more susceptible. Hypotaurine is an intermediate in the catabolism of cysteine to taurine. One of the proposed mechanisms of VPA’s anticonvulsant activity is through enhancing γ-aminobutyric acid (GABA) concentrations within the brain (Johannessen and Johannessen, 2003); and hypotaurine is a known substrate for certain GABA transporter family members, specifically GAT2 (Nishimura et al., 2018). Moreover, deletion of GAT2 has been shown to alter taurine levels in liver and brain tissue (Zhou et al., 2012). Therefore, VPA may interfere with intracellular hypotaurine transport, which may be more detrimental in SWV already having comparatively lower hypotaurine levels.

While that is possible, it is more likely that hypotaurine acts as a protective antioxidant. One of the proposed teratogenic mechanisms of VPA is through oxidative stress induced by several of its toxic ω metabolites. Our metabolomics data support that embryos exposed to VPA were experiencing oxidative stress, based on the ratio of oxidized and reduced glutathione. The antioxidant properties of hypotaurine have been repeatedly demonstrated to elicit protective effects against stress-induced cytotoxicity in several contexts (Fellman and Roth, 1985; Aruoma et al., 1988; Green et al., 1991; Fontana et al., 2004; Fontana et al., 2008). Hypotaurine was shown to protect against oxidative stress in rat placental trophoblasts, while knockout of the gene coding for ezrin, a membrane-cytoskeletal protein necessary for hypotaurine transport, resulted in depleted fetal plasma hypotaurine and reduced fetal weights of knockout embryos (Nishimura et al., 2014; Nishimura et al., 2015). Hypotaurine was also shown to elicit hepatoprotective effects in a rat model of ischemic injury through its antioxidative conversion to taurine (Sakuragawa et al., 2010), and taurine (the downstream product of hypotaurine) was recently shown to reduced hepatic injury in mice after VPA exposure (Khodayar et al., 2021). While the importance of hypotaurine in embryonic development or neural tube closure is not known, the literature suggests it may have protective antioxidant roles.

Supplementation of pregnant dams with 350 mg/kg hypotaurine prior to VPA administration significantly reduced the occurrence of NTD phenotypes. Resorption rates were also lower in the hypotaurine supplemented litters, although this effect was not statistically significant. These data support the hypothesis that enhanced susceptibility to VPA in SWV may be at least partially explained by reduced levels of embryonic hypotaurine. We do not know why hypotaurine levels are lower in SWV embryos compared to C57, and notably taurine was not an indicated MDA in our dataset. It is plausible that genetic variants in the SWV strain may affect synthesis of hypotaurine or its transport between the mother and conceptus. Interestingly, the enzyme responsible for oxidizing hypotaurine during taurine synthesis, flavin-containing monooxygenase 1, was only recently discovered to perform this function in the last few years (Veeravalli et al., 2020). Thus, there are several possible mechanisms that could explain the deficiency of hypotaurine in SWV and each will need to be explored individually.

Pharmacokinetic factors of VPA and the proposed interventions are important to consider. VPA is rapidly metabolized in mice and has a half-life of only .8 h, while its 2-ene-VPA metabolite has a slightly longer half-life of 1.2 h (Nau and Zierer, 1982; Nau, 1985). In adult humans, VPA clearance times range from 10 to 20 h, but pregnancy dramatically affects pharmacokinetics of the drug (Zaccara et al., 1988; Wlodarczyk et al., 2012). While it has been shown that the placenta and certain fetal tissues have the ability to synthesize L-carnitine thanks to low, but detectable expression of the necessary enzymes (Oey et al., 2006), it is primarily transported to the fetus from the maternal circulation via the high affinity carnitine transporter, OCTN2, in the placenta (Shekhawat et al., 2004; Bai et al., 2019). In humans, L-carnitine has a half-life ranging from 2 to 15 h (Goa and Brogden, 1987), and to our knowledge, there is no data available for mice. Hypotaurine is transported across the plasma membrane in cultured rat syncitiotrophoblast cells, and fetal levels of hypotaurine rely on placental hypotaurine transport (Nishimura et al., 2014; Nishimura et al., 2015). Transport of CoA is less studied and poorly understood. While it was traditionally believed that cells exclusively obtain CoA through intracellular biosynthesis, recent evidence suggests there may in fact be mechanisms for CoA uptake in the form of 4′-phosphopantethine, which is produced from CoA by extracellular ectonucleotide pyrophosphatase enzyme activity (Sibon and Strauss, 2016). This may explain why CoA was not an effective intervention in our study. It is also important to note that transport in the placenta during fetal stages of development compared to that of the yolk sac at earlier time points is likely different (Carney et al., 2004). Mechanisms of transport across the vestigial yolk sac include not only transporters, but endocytic mechanisms as well, and expression of specific transporters is poorly studied in yolk sacs at the time of neural tube closure in rodent models. To our knowledge, there is no data on pharmacokinetics of hypotaurine or CoA in mice or humans and neither is currently used in any clinical context. In our study, doses and exposure times for VPA, L-carnitine, and CoA were chosen based on previously reported studies in mice (Wlodarczyk et al., 1996; Nagao et al., 1996). The dose selected for hypotaurine was approximated from a dose shown to attenuate post-ischemic hepatocellular injury in a rat model (Sakuragawa et al., 2010) by converting between rat and mouse equivalent doses (Nair and Jacob, 2016). However, more detailed studies, exploring effective doses and exposure times, as well as maternal and fetal pharmacokinetics could benefit our mechanistic understanding of VPA developmental toxicity and that of other ASMs, as well as mechanisms of effective interventions.

Ultimately, the results of this study may have implications for elucidating the mechanisms of VPA-induced developmental toxicity and assessing overall risk for adverse pregnancy outcomes in women exposed to VPA. By studying this mouse model of differential sensitivity, we were able to identify factors that may contribute to individual susceptibility and target these mechanisms with interventions in the form of carnitine and hypotaurine supplements. One important consideration in determining the efficacy of these supplements as potential mitigation strategies against VPA developmental toxicity is whether these supplements reduce antiseizure properties of VPA. Carnitine supplementation in mice treated with VPA did not reduce anticonvulsant activity (Lammert and Matern, 1997), but additional studies would be needed to test this with hypotaurine. Some other limitations of this study were that the metabolomics experiment, while extremely robust, was an untargeted approach only examining one snapshot of metabolism 2 h after VPA exposure. More targeted flux analyses would be required to truly determine if there are differential pharmacokinetics of VPA in these two strains and measure the dynamic evolution of the metabolomic response to VPA exposure. This is one potential avenue of future study. Additionally, the metabolic factors we identified are specific to the two mouse strains reported on here. Even though embryonic hypoaturine levels appear to contribute to strain-dependent sensitivity of the SWV mice, this likely does not explain the entire spectrum of risk in humans and may not necessarily translate to clinical relevance.

That said, these findings do lay a groundwork supporting future research into genetic or metabolic risk factors associated with VPA and other ASMs in pregnant women. Based on this metabolomics dataset, genetic variants in VPA metabolism, carnitine or fatty acyl metabolism, choline metabolism, methyl metabolism, or cysteine metabolism, and their associated maternal and embryonic transporters may be candidate risk factors for VPA susceptibility. Translational genomic studies are desperately needed to effectively identify such risk-associated genes for adverse outcomes in women and children exposed to VPA or other ASMs. These drugs are critical to the effective management of epilepsy and closely associated neurological diseases affecting millions of women of childbearing potential. Understanding what factors contribute to an individual mother or child’s susceptibility to potential harmful effects will help clinicians more effectively manage these diseases while simultaneously preventing birth defects and other adverse pregnancy outcomes.

## Data Availability

The original contributions presented in the study are included in the article/Supplementary Material, further inquiries can be directed to the corresponding authors.
