# Supplementary material for: Embryonic Hypotaurine Levels Contribute to Strain-Dependent Susceptibility in Mouse Models of Valproate-Induced Neural Tube Defects
Source: Front Cell Dev Biol. 2022 Feb 21;10:832492. doi: 10.3389/fcell.2022.832492 (PMC8898900; doi:10.3389/fcell.2022.832492)
Supplement: Supplementary file 1 [file DataSheet2.PDF]

Supplementary Table Descriptions and Figure Legends

Supplementary Table 1. List of metabolites of differential abundance (MDAs) (FDR < 0.05) in SWV and C57 embryos with and without VPA exposure at E8.5 (600mg/kg). Relevant statistics, such as adjusted p-values and fold changes are reported in the columns. Metabolites with increased abundance are highlighted in blue, while metabolites of decreased abundance are highlighted in red.

Supplementary Table 2. List of metabolites of differential abundance (MDAs) (FDR < 0.05) between vehicle control C57 and SWV embryos at E8.5. Relevant statistics, such as adjusted p-values and fold changes are reported in the columns. Metabolites with increased abundance are highlighted in blue, while metabolites of decreased abundance are highlighted in red.

Supplementary Table 3. List of metabolites of differential abundance (MDAs) (FDR < 0.05) between vehicle control C57 and VPA-exposed (600mg/kg) C57 embryos at E8.5. Relevant statistics, such as adjusted p-values and fold changes are reported in the columns. Metabolites with increased abundance are highlighted in blue, while metabolites of decreased abundance are highlighted in red.

Supplementary Table 4. List of metabolites of differential abundance (MDAs) (FDR < 0.05) between vehicle control C57 and VPA-exposed (600mg/kg) SWV embryos at E8.5. Relevant statistics, such as adjusted p-values and fold changes are reported in the columns. Metabolites with increased abundance are highlighted in blue, while metabolites of decreased abundance are highlighted in red.

Supplementary Figure 1. Occurrence of NTDs by embryonic sex in VPA-treated SWV. Proportion of female and male embryos with exencephaly phenotypes in SWV litters exposed to 600mg/kg VPA. **Statistical significance determined by two-sample proportion test ($\alpha < 0.05$).*

Supplementary Figure 2. Number of increased and decreased MDAs in each super and sub pathway classification. Number of increased and decreased metabolites of differential abundance (FDR < 0.05) in super and sub pathway classifications between VPA-exposed (600 mg/kg) SWV and C57 embryos compared to vehicle control embryos.

Supplementary Figure 3. Abundance of metabolites involved in the methylation cycle and SAM/SAH ratio. (Top panels) Peak intensities for methionine, s-adenosylmethionine (SAM), s-adenosylhomocysteine (SAH), and homocysteine in SWV and C57 embryos with and without VPA exposure at E8.5 (600mg/kg). (Lower panels) Homocysteine/Methionine ratio and inverse SAM/SAH ratio (i.e. SAH/SAM) in SWV and C57 embryos with and without VPA exposure at E8.5 (600mg/kg). **Statistical significance determined by one-way ANOVA with Dunnett's multiple comparisons ($\alpha < 0.05$).*

Supplementary Figure 4. Ratio of oxidized to reduced glutathione. Ratio of oxidized glutathione (GSH) to reduced glutathione (GSSG) in SWV and C57 embryos with and without VPA exposure at E8.5 (600mg/kg) as a measure of oxidative stress. **Statistical significance determined by one-way ANOVA with Dunnett's multiple comparisons ($\alpha < 0.05$).*

Supplementary Figure 5. Carnitine abundance in embryos. Abundance of L-carnitine (peak intensity) in SWV and C57 embryos with and without VPA exposure at E8.5 (600mg/kg). **Statistical significance determined by one-way ANOVA with Dunnett's multiple comparisons (α < 0.05).*
