# Supplementary material for: Embryonic Hypotaurine Levels Contribute to Strain-Dependent Susceptibility in Mouse Models of Valproate-Induced Neural Tube Defects
Source: Front Cell Dev Biol. 2022 Feb 21;10:832492. doi: 10.3389/fcell.2022.832492 (PMC8898900; doi:10.3389/fcell.2022.832492)

Supplementary Figure 1. Occurrence of NTDs by embryonic sex in VPA-treated SWV.

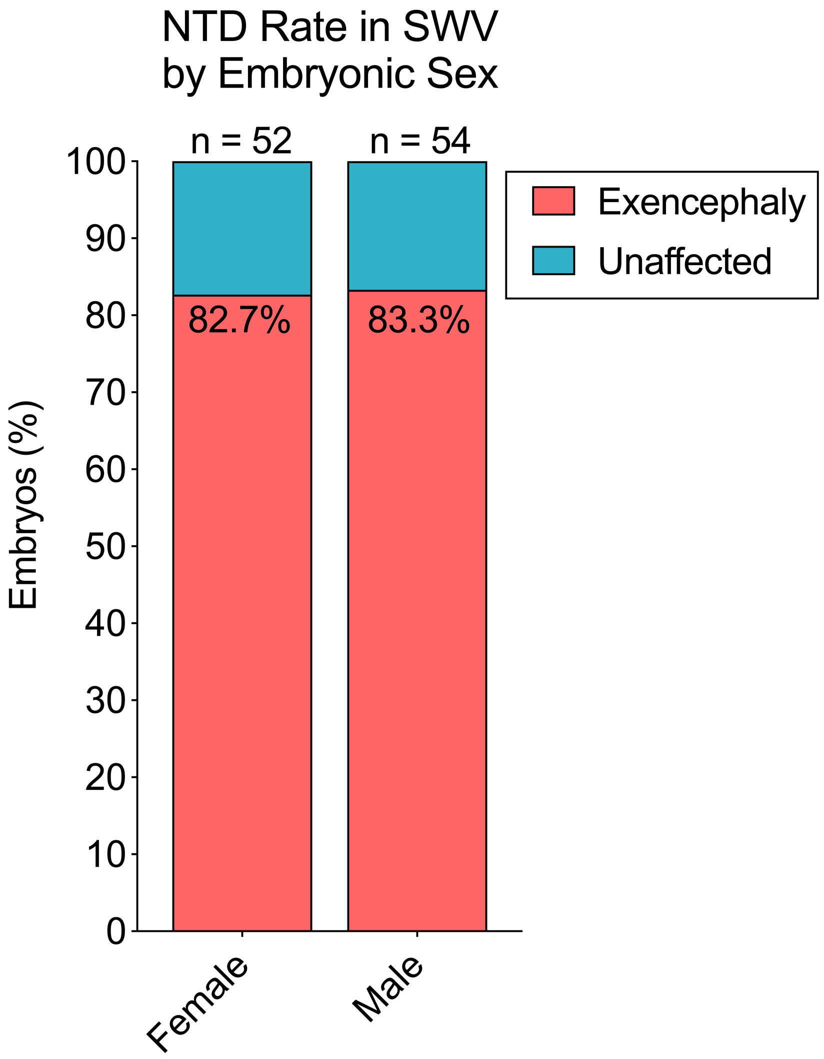

Supplementary Figure 2. Number of increased and decreased MDAs in each super and sub pathway classification.

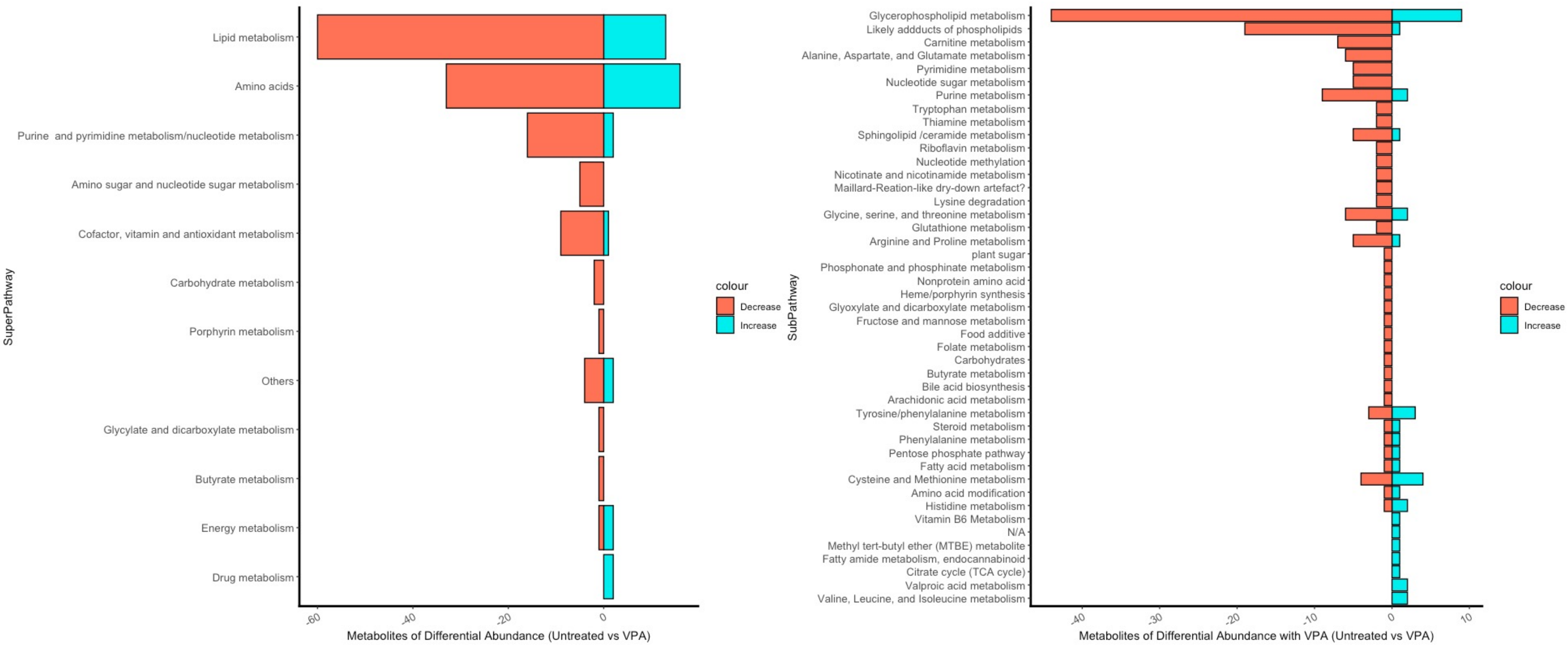

Supplementary Figure 3. Abundance of metabolites involved in the methylation cycle and the SAM/SAH ratio.

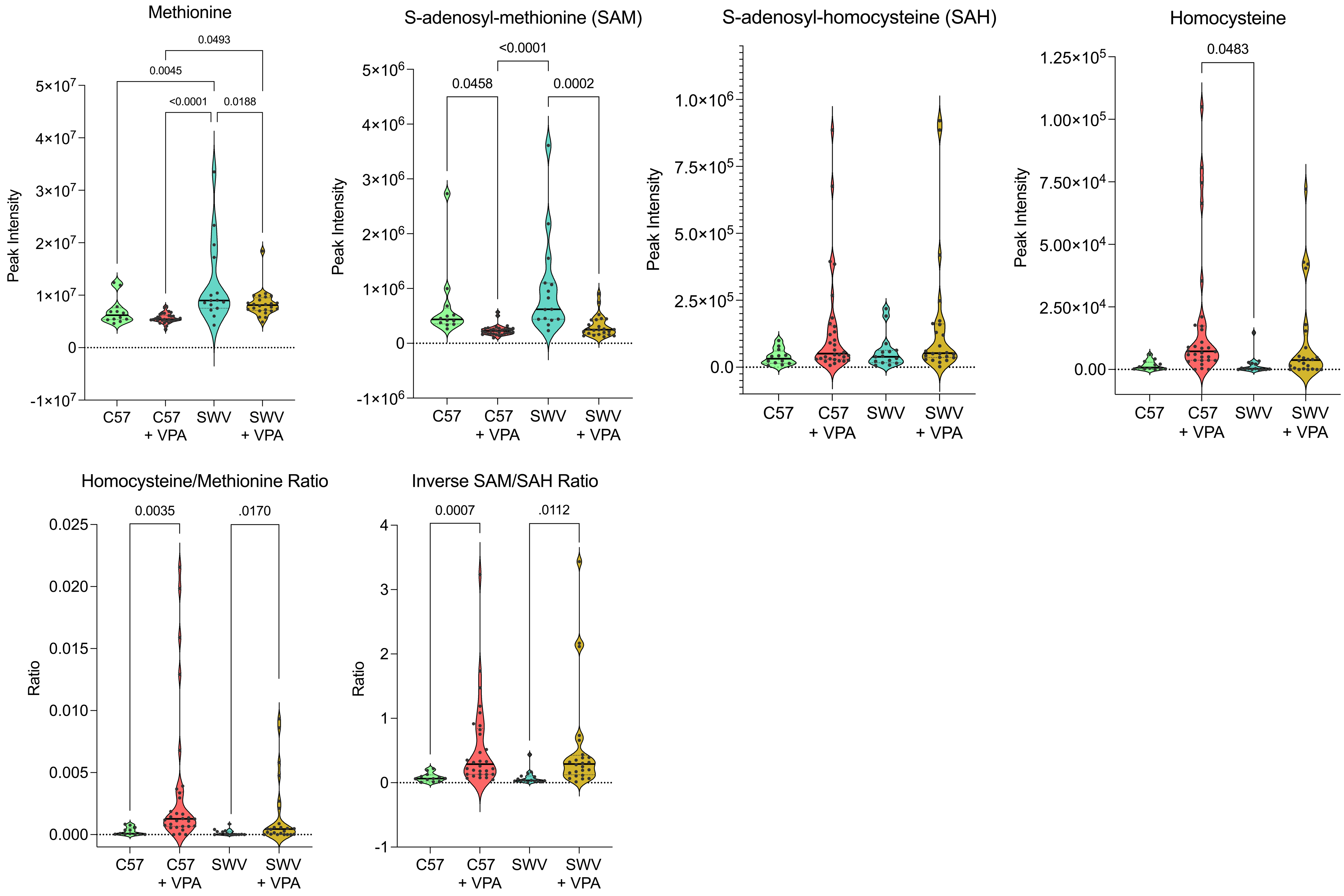

Supplementary Figure 4. Ratio of oxidized glutathione to reduced glutathione.

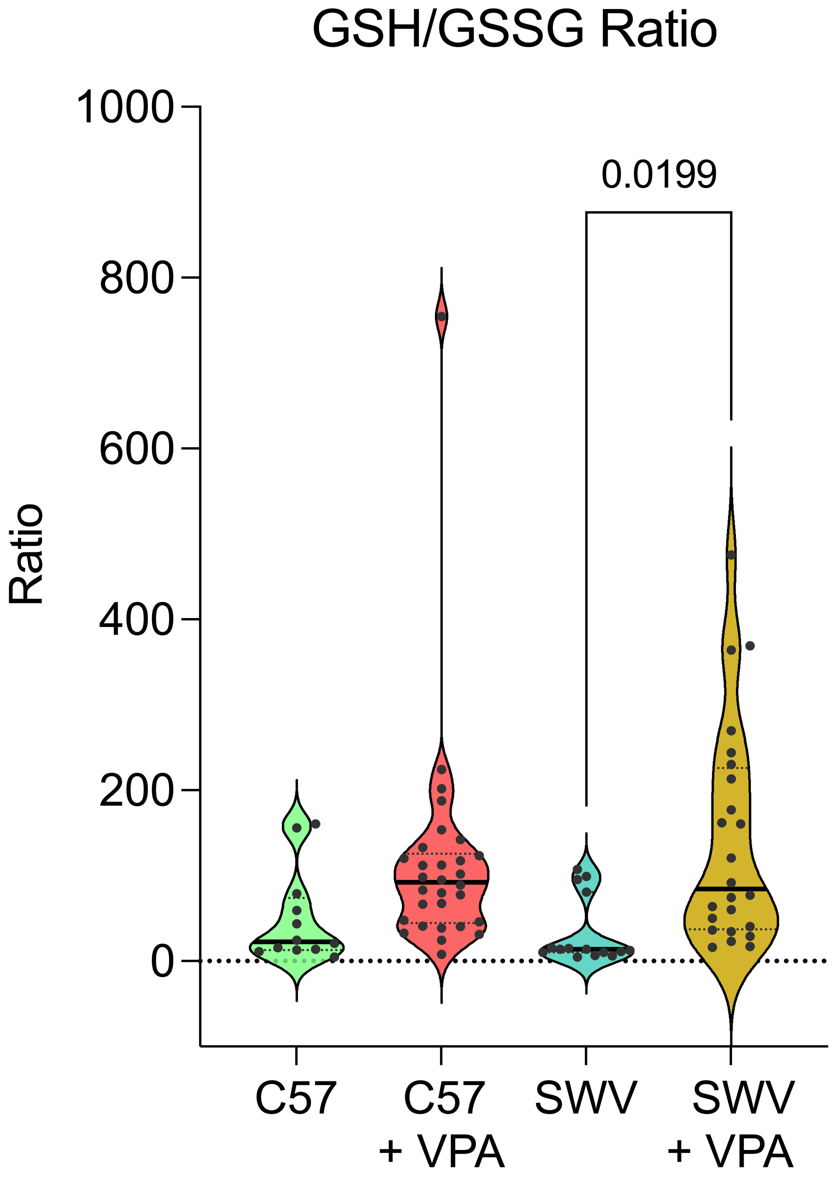

Supplementary Figure 5. Carnitine abundance in embryos.

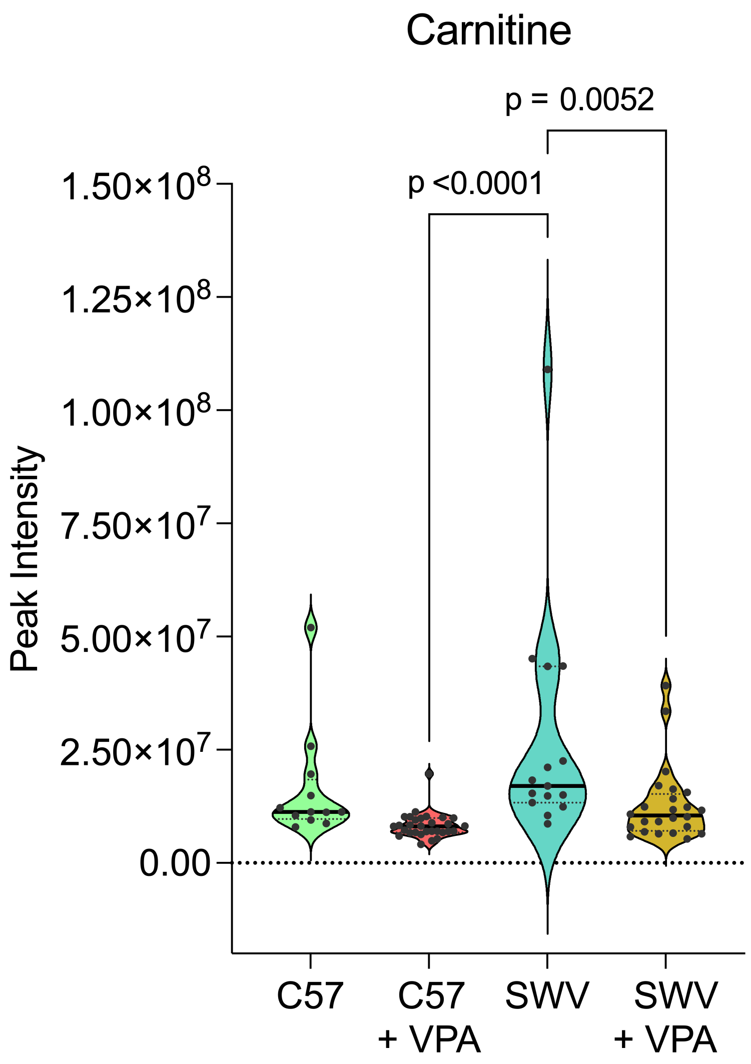

Supplement: Supplementary file 4 [file Presentation1.PDF]
